# Legislation and policy for involuntary mental healthcare across countries in the FOSTREN network: rationale, development of mapping survey and protocol

**DOI:** 10.1192/bjo.2024.744

**Published:** 2024-09-19

**Authors:** Deborah Oyine Aluh, Tella Lantta, Tânia Lourenço, Søren Fryd Birkeland, Giulio Castelpietra, Jovo Dedovic, José Miguel Caldas-de-Almeida, Jorun Rugkåsa

**Affiliations:** Lisbon Institute of Global Mental Health, Comprehensive Health Research Centre (CHRC), NOVA Medical School, NOVA University of Lisbon, Portugal; and Department of Clinical Pharmacy and Pharmacy Management, University of Nigeria Nsukka, Nigeria; Department of Nursing Science, University of Turku, Finland; and Centre for Forensic Behaviour Sciences, Swinburne University of Technology, Australia; Center for Health Technology and Services Research (CINTESIS@RISE), NursID – Innovation & Development in Nursing, Portugal; and Nursing Department, Escola Superior de Enfermagem de São José de Cluny, Portugal; OPEN Research Unit, Odense Universitetshospital, Denmark; and Department of Regional Health Research, University of Southern Denmark, Denmark; Community Psychiatry Service, Department Adult 2, Centre Neuchâtelois de Psychiatrie, Switzerland; Addiction Department, Special Psychiatric Hospital Dobrota Kotor, Montenegro; Lisbon Institute of Global Mental Health, Comprehensive Health Research Centre (CHRC), NOVA Medical School, NOVA University of Lisbon, Portugal; Health Services Research Unit (HØKH), Akershus University Hospital, Norway; Department of Mental Health Care, Faculty of Health Sciences, Oslo Metropolitan University, Norway; and Centre for Care Research, University of South-Eastern Norway, Norway

**Keywords:** Coercion, mental health legislation, policies, Europe, United Nations Convention on the Rights of Persons with Disabilities

## Abstract

**Background:**

Several countries are currently revising or have already revised their mental health laws to align with the global movement to reduce the use of coercive care. No government has yet fully implemented the recommendation of the United Nations Convention on the Rights of Persons with Disabilities (UNCRPD) to eliminate the use of coercion in mental healthcare. Consequently, the international field of mental health law and policy is in a degree of flux.

**Aims:**

To describe the rationale, development and protocol for a project that will map and examine how mental health laws, policies and service capacity across European countries relate to the use of coercive measures, including involuntary admissions and treatment, restraints and seclusion. This will help to better understand the current situation and explore future directions of policies regarding coercive care.

**Method:**

The project is being carried out under the purview of the European Cooperation in Science and Technology (COST) action network, entitled FOSTREN (Fostering and Strengthening Approaches to Reducing Coercion in European Mental Health Services). A multidisciplinary group of experts developed a comprehensive survey assessing mental health laws, policies and service frameworks, based on World Health Organization and UNCRPD recommendations. The survey was piloted in three countries, revised and disseminated to 30 FOSTREN country representatives. The survey will provide data for three strands of work on legislation, policies and service-level context. A comprehensive evaluation will be conducted, drawing on findings from all work packages.

**Conclusions:**

The project could inform the development of strategies, interventions and legislation to address gaps and promote compliance with international standards.

Coercion in mental healthcare has been defined as ‘forceful actions, involuntary treatment, or threats undertaken while providing treatment or addressing perceived harm that a person poses to themselves or others’.^1^ Coercive care is codified in law, and typically includes involuntary admission, outpatient commitment, mechanical restraint, chemical restraint and seclusion. Coercion conflicts with central ethical principles of healthcare: autonomy and the right to self-determination.^2–4^ Several human rights documents and patient rights charters support patients’ involvement in making decisions about their treatment, a right that in many countries is enforced through a legal requirement for informed consent before healthcare interventions.^5,6^ The right to self-determination implies that patients can voice their opinion and are given the opportunity to choose what intervention to undergo. Exercising this right is considered a cornerstone to meeting patients’ needs and expectations.^7^ Patient-centred care that is respectful of individual preferences, needs and values is among the six aims for quality improvement for 21st-century healthcare systems recommended by the US Committee on the Quality of Health Care.^7^ Patient-centeredness intrinsically entails giving patients ‘the opportunity to exercise the degree of control they choose over health care decisions that affect them’.^7^ With these approaches as a backdrop, the topic of coercion is a controversial one. This controversy has grown in recent times as there is growing evidence of the negative effects associated with its use.^8^

The United Nations Convention on the Rights of Persons with Disabilities (UNCRPD), which was developed in 2006 and came into force in 2008, is the most comprehensive legal document to date that outlines the rights of individuals with disabilities, including those with mental health conditions.^9^ It requires signatories to amend their mental health laws to ensure the protection and promotion of all fundamental human rights of individuals with disabilities, including those with mental health conditions. The UNCRPD places great importance on addressing coercion in mental healthcare through several key principles. Articles 12 and 25 emphasise autonomy, informed decision-making and free informed consent, discouraging practices such as forced treatment or involuntary admission. Article 12, titled ‘Equal recognition before the law’, highlights the right to exercise legal capacity (through support if required) and independence of individuals with disabilities, ensuring their decision-making is free from undue influence or coercion. Article 14 asserts that having a disability cannot be used as a ground for denying the fundamental right to liberty, thereby reinforcing the protection against unjustified detention for persons with disabilities. Additionally, Articles 13 and 19 of the UNCRPD focus on providing support, alternatives and legal protection for individuals with disabilities. Article 13 guarantees efficient access to the legal system and safeguards their rights, whereas Article 19 advocates against institutionalisation and promotes the development of community-based health services and support. Although not explicitly stated, the principle of the least restrictive alternative permeates the UNCRPD, especially regarding the right to live independently and participate in society. The convention also includes safeguards against abuse and torture, primarily addressed in Article 15, which protects individuals from any form of mistreatment and emphasises the importance of prompt and unbiased investigations.^3^ These principles collectively advocate for a human rights-based approach to mental healthcare, prioritising the respect for autonomy, dignity and well-being of individuals with disabilities, including people with mental health conditions (PMHCs).

According to the interpretation of the UNCRPD by the United Nations Committee on the Rights of Persons with Disabilities, all forms of substituted decision-making, which often occur when individuals are considered to lack ‘insight’, are seen as a breach of the Convention's assurance of equal right to exercise legal capacity. By implication, the committee argues, any form of involuntary treatment is forbidden.^10^ This interpretation has faced criticism from various United Nations bodies, governments, researchers, practitioners and users, who argue that this absolutist interpretation of the UNCRPD could unintentionally deprive some individuals of their right to health and increase the risk of harm by denying necessary treatment during emergencies or situations where they pose a danger to themselves or others.^5,11^ The differing interpretations of the UNCRPD led to the well-known ‘Geneva Impasse’. On one side, supported by the Special Rapporteur on Disability, the first Special Rapporteur on the Rights of Persons with Disabilities, the United Nations Working Group on Arbitrary Detention and the United Nations High Commissioner on Human Rights, the UNCRPD committee argues for an absolute prohibition of involuntary detention and treatment.^12^ On the other hand, the United Nations Human Rights Committee and the Subcommittee on Prevention of Torture and Other Cruel, Inhuman, or Degrading Treatment or Punishment do not support the UNCRPD's strict interpretation.^13,14^

Although various ethical arguments are presented for and against the elimination of coercion in mental healthcare,^15^ there is a consensus on the need to reduce their use to the absolute minimum. Countries are facing challenges in aligning their national mental health legislation with the approach advocated by the UNCRPD. One particularly contentious issue is the Committee's stance on freedom from involuntary admissions, which has led to many countries to disregard their recommendations.^14^ No government has fully implemented the UNCRPD's proposal, and coercive measures are widely used in many countries. However, several countries are in the process of, or have already, revised their mental health laws to make them better aligned with the global movement to reduce the use of coercion in mental healthcare. Consequently, the international field of mental health law and policy is in a degree of flux.

In this article, we present the background and protocol for a comprehensive project examining the current situation and future direction of mental health legislation and policy related to coercion in mental healthcare across Europe. We first summarise existing research on this topic to identify the gaps that provide the rationale for conducting the current study. We then describe the methodology employed to design and conduct the study, before sketching out areas for analysis.

## Existing research and knowledge gaps

We reviewed the literature to identify key studies on mental health laws and policies since the year 2000. As involuntary admissions are often the gateway to the use of other coercive measures,^16^ we selected comprehensive studies and reviews that mostly focused on involuntary admissions.

### Comparing the mental health legislation of Commonwealth countries

Given the diversity and complexities associated with mental healthcare systems and legislation, there have been limited studies conducted to compare and assess laws and policies across countries. Very few of these studies have been conducted outside Europe. The criteria for involuntary treatment in 32 Commonwealth Mental Health Acts were explored and compared by Fistein et al^17^ in 2009, using a framework developed from standards derived from the Universal Declaration of Human Rights. They found widespread deviation from standards, suggesting that some of the laws may not have adequately protected the human rights of people with mental disorders. The identified trends in mental health law reform included broad diagnostic criteria, capacity and treatability tests, treatment in the interests of health rather than safety, and regular treatment order reviews. They also found considerable variation in the criteria for involuntary admission and treatment, which were attributed to differing value perspectives, failure to keep up with changing attitudes toward mental disorder and variations in available treatment resources and law reform.^17^ Since 2009, subsequent studies or updates have not been conducted to assess changes or improvements in compliance with human rights standards.

### Comparative studies of mental health legislation in selected jurisdictions

In 2017, a comprehensive analysis was conducted to explore the similarities and differences in mental health legislation across five jurisdictions: the Republic of Ireland, England and Wales, Scotland, Ontario (Canada) and Victoria (Australia).^18^ The focus of the examination was on the process of involuntary admission, the review of Admission Orders and the legal procedures concerning treatment in the absence of patient consent. They found that although all jurisdictions allowed for the detention of individuals with mental disorders, the definition of mental disorder varied among these regions. Moreover, there were several additional distinctions among the five jurisdictions, such as the length of time before an independent review of involuntary detention and the significance of supported decision-making.^18^ Another review, conducted in 2019, examined legislation and associated issues from four diverse South Asian countries (Bangladesh, India, Pakistan and Sri Lanka) with a British colonial past and a previous Lunacy Act of 1845.^19^ The mental health law assessment was largely based on the World Health Organization checklist for mental health legislation and focused on the criteria and process for involuntary detention of PMHCs. They found that relevant legislation had evolved differently in each of the four countries. Each country faced challenges when reforming or implementing its mental health laws. Barriers included legal safeguards, human rights protections, funding, resources, the absence of a robust wider health system, political support and suboptimal mental health literacy.^19^ A recent study compared the legal framework of mental health law and compulsory hospital admission in Italy and the UK. By reviewing each country's latest amendments to mental health law and the number of compulsory hospital admissions, the study aimed to understand the impact of changes in mental healthcare. The data revealed rising detention rates in the UK, with a disproportionate use of the legal framework among people from Black and minority ethnic groups. In Italy, compulsory admissions were lower, but have been increasing in recent years. However, due to a lack of national data on ethnicity, understanding of compulsory admission, discrimination and stigma in mental health was limited.^20^ The impact of these legal differences on coercive measures in these jurisdictions were not discussed, leaving a gap in understanding the practical effects of varying legislations.

### Coercive measures in mental healthcare across Europe and globally

An international comparative study by Dlouhy^21^ in 2014 aimed to describe and compare the mental health policies in seven Eastern European countries with a shared communist history. The key findings were that the transition in the 1990s led to the formulation of new mental health policies and legislation, emphasising patients’ human rights and the balance between community and hospital services, and that mental health services were funded through public health insurance, with no separate budget. The influence of totalitarian history continued to affect social and economic life, hindering the achievement of a balanced mental health system.^21^

The EUNOMIA project, funded by the European Commission, aimed to evaluate the clinical practice and outcomes of coercive measures in 12 European countries.^16^ Although it did not specifically focus on mental health legislation, the study proved valuable in illustrating variations in the use of coercive practices among European countries. The percentage of patients subjected to coercive measures varied from 21 to 59% across the countries. They found that coercive measures were employed in a significant number of involuntarily admitted patients throughout Europe, and that their use seemed to be influenced by the diagnosis, illness severity and the cultural norms and clinical traditions of each individual country. The primary reason for implementing these measures was patient aggression toward others. In eight countries, forced medication was the most used coercive measure, whereas mechanical restraint was predominant in two countries.^16^ Similarly, a recent study that compared coercion rates and their median duration across nine countries across four continents, using a standardised measure, found significant variability in these factors, even after controlling for the national population.^22^ The knowledge gap in understanding the relationship between the specific legal frameworks of the countries studied and the rates and types of coercion used persists, especially given the significant variability in coercion rates and durations across countries.

### Impact of legislation on involuntary admission rates

Although the use of coercive measures in mental healthcare may be seen as a reflection of the underlying characteristics of mental health legislation and policies in a specific jurisdiction, studies of the impact of legislation on the rates of involuntary admission have yielded inconsistent results. The search for the most effective legal framework that can protect the rights of individuals with mental health conditions and the wider public while minimising coercion remains uncertain. Limited research has explored the relationship between the rates of coercive measures in countries with specific aspects of the legal criteria and procedures for commitment. In a study conducted by Salize and Dressing,^23^ the relationship between mental health legislation and involuntary admission rates in 15 countries in the European Union was examined. It was found that all European Union Member States require individuals to have a diagnosed mental health condition for detention, with additional criteria such as potential harm to oneself or others being common. There were no significant differences in rates of involuntary admissions between countries that used the ‘danger’ criterion and those that used the ‘need for treatment’ criterion. Furthermore, there were no significant differences between countries where non-medical authorities, such as judges, prosecutors, mayors or entities unrelated to the medical system, made the final decision on involuntary admission and countries where psychiatrists or other medical specialists made that decision. The study indicated a tendency toward lower involuntary admission rates in countries where a legal representative was involved, suggesting the need for further investigation.^23^ A recent and extensive comparison of annual rates of involuntary admission in 22 countries found that there was no connection between the characteristics of the legal framework and these rates. Instead, the rates appeared to be influenced by external factors such as the level of absolute poverty, gross domestic product and per capita healthcare spending, the percentage of foreign-born individuals in the population and the number of in-patient beds available.^24^ The European Psychiatric Association's Ethics Committee recently conducted a survey on involuntary admission procedures for patients with mental disorders in 40 countries. The survey, which included 44 national psychiatric associations, aimed to identify similarities and differences in legal and medical procedures regarding compulsory admissions. The report discussed the involvement of non-medical individuals such as legal counsels and civil authorities in the involuntary admission process across various European countries. It also highlighted a shift toward using the need for treatment criterion as opposed to the commonly used, but criticised, danger criterion.^25^ Although the survey was useful in providing a portrait of compulsory admission process in European countries, the use of only dichotomous responses limited its ability to provide a comprehensive understanding of the mental health laws. Furthermore, issues related to involuntary treatment in the community and other coercive measures (e.g. restraints) were not explored.

## Rationale for the study

Considering the evolving landscape of mental healthcare and, within that, the rights of PMHCs, it is crucial to address and fully understand the legal frameworks and policies related to coercive measures used in psychiatric treatments. By psychiatric treatments, we refer to both civil and forensic mental healthcare in in-patient and community settings. Overviews of these frameworks and policies are scarce, particularly within Europe, where much has changed since the turn of the century. Some of the existing scholarly works, like governmental reports, are written in languages other than English. These documents often do not conform to the rigorous standards of scientific research and, unfortunately, remain untranslated, thus creating a language barrier to their broader dissemination. The most comprehensive study evaluating the mental health legal frameworks of European countries predated the UNCRPD and did not consider new member countries that have joined the European Union since then. As a result, there is an evident need for an updated study that not only considers recent developments regarding the rights of PMHCs, but also investigates other coercive measures beyond involuntary admissions. An exclusive focus on involuntary admissions does not provide a holistic understanding of how legal factors affect mental healthcare. Acknowledging and analysing the complexity behind the use of coercive measures necessitates an examination of contextual variables on various levels. Simply understanding legal factors alone may fail to provide an accurate depiction of how these measures are implemented and influenced by extenuating circumstances. For example, policies, guidelines and service-level factors must be considered because of their potential overlaps with legal frameworks. By conducting an extensive mapping exercise that builds on previous studies’ limitations, we will gain valuable insights into how legal frameworks operate in conjunction with other influential factors.

## Aims

The project we describe in this article aims to map current mental health laws, policies and service context-related involuntary admissions and treatment, restraints and seclusion in 30 European countries. The survey will also include planned policies, which will form basis for an analysis of the future intended direction of coercive mental healthcare in these countries.

## Method

The project is being carried out under the purview of the European Union European Cooperation in Science and Technology (COST)-funded network initiative, FOSTREN.^26^ FOSTREN, which stands for Fostering and Strengthening Approaches to Reducing Coercion in European Mental Health Services, is a consortium of professionals, patients and researchers who are dedicated to understanding methods for effectively shifting services away from coercive practices such as seclusion, restraint and involuntary admission, toward more cooperative care approaches. The network is open to all 41 COST member countries, including Israel and South Africa, who are cooperating and partner members, respectively.^27^ Thirty of these countries have joined FOSTREN and have representation on the Management Committee,^28^ and were the countries considered in this survey. They include Austria, Belgium, Bosnia and Herzegovina, Bulgaria, Croatia, Czech Republic, Cyprus, Denmark, Estonia, Finland, France, Germany, Hungary, Ireland, Italy, Israel, Latvia, Malta, Moldova, Montenegro, The Netherlands, North Macedonia, Norway, Poland, Portugal, Romania, Slovakia, Slovenia, Spain, Sweden, Switzerland, Turkey and the UK. The methodology employed will be described in subsequent sections.

### Forming a core group of experts

The primary aim of the project was to establish a comprehensive repository containing information on legislation, policies and service frameworks related to coercion within the 30 FOSTREN countries. Members from FOSTREN who expressed interest were co-opted into the research team, with a focus on ensuring representation from diverse European regions. The team included mental health practitioners, a sociologist, a legal practitioner and researchers possessing a range of professional experience levels. Within this group, a core group of three individuals was designated to lead the project, reporting back to the larger group at regular intervals.

### Development of survey instruments

To commence the development of the survey instrument, a meeting was convened in February 2022, where the overall objectives and necessary tasks were discussed. An extensive review of the literature was undertaken, and previous reports, documents and white papers pertaining to mental health laws and policies were gathered. The most comprehensive instrument available was the World Health Organization's (WHO) ‘Mental Health Policy and Service Guidance Package–Mental Health Legislation & Human Rights’,^29^ which was formulated before the UNCRPD. Considering the UNCRPD recommendations and based on the framework of the WHO Checklist on Mental Health Legislation, a fresh set of questions was formulated (see Supplementary Material available at https://doi.org/10.1192/bjo.2024.744).

The survey was divided into three sections. Section 1 consisted of 74 questions on the legislation in each country concerning the treatment of individuals with mental disorders. This included items on involuntary admissions, involuntary treatment in the community, forensic and civil law compulsion, and specific coercive measures like seclusion, mechanical and chemical restraints. As we aimed to examine legislation in the context of the UNCRPD, we made it clear that we were focused on the letter of the law, rather than its practical interpretation. Section 2, consisting of nine questions, sought information about specific policies issued since 2005 regarding the use of involuntary care and/or reduction in the use of such care, and the positions of various stakeholders within each country regarding coercion in mental healthcare. It further asks about what is in the pipeline regarding such policies in the country. Section 3, consisting of eight questions, aimed to map the scope and capacity of mental health services (in-patient and out-patient) in each country. Additionally, it sought to gather information on the availability of mental health professionals and rates of involuntary admission.

To ensure clarity, a glossary of terms was developed to provide definitions for the terminology and concepts used in the survey. The creation of this glossary was of great importance because of the vast diversity across countries in terms of linguistic, cultural and care traditions, which makes it challenging for stakeholders to intuitively understand the terminology used to describe coercive practices across contexts.

For ease of response and analysis, most questions were formulated to be close-ended with a range set of options, and with an option of ‘not obtainable’ when specific information was not accessible. Respondents were also prompted to specify additional details in cases of unique circumstances or when the none of alternative answers fitted their context. Although we only sought one set of responses from each country, respondents were encouraged to collaborate with relevant professionals, experts and their respective health authorities to complete the survey.

### Pilot and revision

To assess the comprehensibility, feasibility and time needed to complete the survey, it was initially tested in Portugal, Finland and Montenegro. These countries were deliberately chosen to account for the variation in mental health systems across different regions of Europe. The pilot survey provided valuable insights that were used to make necessary adjustments to the survey and refine the instructions given to the respondents. A decision was made to specify that we wanted answers adhering strictly to the ‘letter of the law’ rather than the ‘spirit of the law’, as the latter would open for a degree of legal interpretation. Respondents were thus urged to focus on the technical language of the law rather than its various interpretations in clinical practice. They were also prompted to provide comments in cases where there was a notable discrepancy between the actual wording of the law and its customary interpretation or application. During the piloting phase, the involvement of psychiatrists who regularly apply the law proved highly beneficial in compiling information on legislation. Additionally, the input and collaboration of multiple individuals were invaluable in addressing any uncertainties and ensuring compliance with the focus on letter of the law, rather than subjective interpretations. Gathering information on the scope of services varied significantly between countries in the pilot survey, with some relying on publicly available routine statistics and others requiring specific requests to their Ministry of Health. Thus, for questions related to policy, the involvement of individuals working in national bodies such as ministries or directorates, as well as researchers with a focus on policy work, was encouraged. The revisions following the pilot were finalised in June 2023, and the questionnaires that will be used in the study can be found in the Supplementary Material.

Despite the revisions made to the final versions, additional issues may arise when data is collected from a broader range of countries. These potential problems will be addressed in future publications.

### Procedure and participants

The questionnaire was disseminated to the management committee members of the FOSTREN network, who are representatives of their respective countries involved in the COST action. The survey, which was in the English language, included an introduction letter and an acknowledgement form, and was sent to the two Management Committee members from each of the 30 FOSTREN countries. The Management Committee is the formal management structure of the action. Two members are nominated from each country by their respective COST national coordinator, according to national procedures. The committee is responsible for making important administrative decisions, such as approving the annual budget and considering new countries wishing to join the action. FOSTREN Management Committee members include professionals from various fields, including academia, psychiatry, mental health nursing and psychology. They were requested to identify and collaborate with relevant networks in their countries that represent professional groups involved in the implementation of mental health laws, such as medical practitioners, mental health nurses and criminal justice/legal professionals. To ensure a comprehensive collection of data, reminders were periodically sent to stakeholders, and the submission deadline has been extended.

### Quality control

The survey was explicitly stated to require a team effort, discouraging individual completion. It was recommended that management committee members establish and coordinate a group of stakeholders from their respective countries, including representatives from the Ministries of Health or relevant authorities. Troubleshooting meetings were offered by the core group to address any doubts or queries. To assess the reliability and validity of the collected information, respondents were required to provide links to the websites where the information was sourced.

### Data management and analysis

Following data entry and quality checks, the first stage of the analysis will be organised in three work strands on the legislative, policy and service context, respectively, to provide overview. We will then draw on all three data sources as necessary to conduct comprehensive, in-depth analyses on selected topics of care related to the reduction of coercion. Detailed analysis plans will depend on the completeness and quality of the final data-set, but these topics likely include care planning approaches, seclusion/restraints practices, patient involvement, community treatment orders, forensic services and future policy. Both quantitative and qualitative methods will be used. The quantitative variables will be analysed using descriptive and inferential statistics. For the textual components of the data, qualitative content or thematic analysis will be employed as appropriate,^30^ and policy documents will be subject to relevant policy^31^ or document analyses.^32^ Data analyses and interpretations will be conducted in collaboration with patients. We will also produce, toward the end of the study, a comprehensive report that will draw on all findings. This will aim to include the service-level context in each country, to provide a comprehensive overview of involuntary care *vis-à-vis* the UNCRPD in the FOSTREN countries. The data collected will be placed in a publicly available repository at the end of the study period. An updated version of the study instrument will also be publicly available for countries or regions seeking to evaluate their mental health laws, policies and services.

### Ethical considerations

The study is hosted at the Akershus University Hospital, Norway. It received approval from the Hospital Privacy Ombudsman (reference 2023_85), following the decision of the South-Eastern Research Ethics Committee that it falls outside the scope of the Norwegian Health Research Act (reference 616738). The names and contact details of the collaborators will be securely stored on the hospital's server and will not be disclosed.

## Discussion

The mapping survey has the potential to significantly affect mental health laws, policies and services in Europe. Utilizing a systematised instrument created based on UNCRPD and WHO recommendations ensures that the evaluation process aligns with internationally recognized standards and best practices. This approach increases the credibility and reliability of the analysis. Mapping can also facilitate the identification of areas where mental health laws, policies and services are in line with UNCRPD recommendations and where further improvement is required. It provides a basis for developing strategies and interventions to address gaps and promote compliance with international standards. Mapping mental health laws, policies and services can help to identify gaps and inconsistencies in the current system. By studying the variations and similarities across different European countries, policy makers can gain insights into areas where improvements or harmonisation is needed. Through mapping, policy makers can identify countries that have successful mental health laws, policies and services. By benchmarking these best practices, decision makers can learn from successful models and implement effective strategies in their jurisdictions. Continuous evaluation and monitoring of the implemented changes are also essential. By regularly evaluating mental health laws, policies and services, areas that require further improvement will be identified. This proactive approach will facilitate ongoing advancements and ensure that the mental health landscape in Europe is constantly evolving for the better.

The main challenge anticipated in the analyses and interpretation of survey responses is the fragmentation and inconsistency across different European countries in terms of mental health laws, policies and services. A recent global comparison of coercive practices has shed light on the highly variable, poorly reported and inadequately reported nature of coercive practices across countries.^22^

### Strengths and limitations

A significant strength of the mapping study lies in the active participation of a diverse group of multidisciplinary researchers from different countries with varied mental health systems. The systematic development of the study instrument, guided by the UNCRPD and WHO recommendations, facilitates a standardised comparison and thorough analysis of complex legislative frameworks across Europe. The comprehensive data collection on mental health laws, policies and service-level variables gives it an advantage over previous studies. However, a limitation arises from the linguistic complexity of designing the instrument in English, as most mental health laws are available in European languages other than English, which may result in some loss of nuanced meaning during translation and data completion. Additionally, the absence of patient involvement within the research team and the FORSTREN network (and, consequently, in developing the survey) are noteworthy limitations, given the importance of incorporating the perspectives of individuals with mental health conditions into research that directly concerns them. Inviting patients to take part in data analysis and interpretation will enhance the relevance and impact of our work. It should also be noted that the availability and reliability of data on mental health services may vary greatly among the different countries.

In conclusion, conducting a comprehensive mapping of mental health laws, policies and services in Europe, as described here, can provide new valuable knowledge to inform decision makers and the development of best practice guidelines for services and clinicians. It can help identify specific areas for improvement, support evidence-based decision-making and promote collaboration among all stakeholders to work toward the shared aim of reducing the use of coercive mental healthcare.

## Supporting information

Aluh et al. supplementary material Aluh et al. supplementary material

## Data Availability

Data availability is not applicable to this article as no new data were created or analysed in this study.
