# Supplementary material for: Legislation and policy for involuntary mental healthcare across countries in the FOSTREN network: rationale, development of mapping survey and protocol
Source: BJPsych Open. 2024 Sep 19;10(5):e154. doi: 10.1192/bjo.2024.744 (PMC11457212; doi:10.1192/bjo.2024.744)
Supplement: Aluh et al. supplementary material [file S2056472424007440sup001.docx]

**FOSTREN LAW AND POLICY PROJECT**

**GLOSSARY OF TERMS**

The terminology used in describing and researching involuntary care varies across countries and studies. Given the broad range of languages we collectively operate with, translation of terminology is sometimes tricky.

To ensure consistency in the data collected, we have developed this glossary of how the various terms should be understood for the purposes of the FOSTREN Law and Policy Project. In the different parts of the research instrument, we have marked relevant terms with an asterisk (*), to indicate that you may refer to this glossary if you are uncertain about what is meant by a question or how to interpret a term.

**Advance directives**: refers to a written statement by a patient when well, which sets out the way in which they want to be treated and/or treatment they do not want for their mental health condition should they deteriorate.

**Care Planning Approach**: CPA is a structure for ensuring a person’s package of care is planned, delivered, reviewed and coordinated around individual needs. Often this is the responsibility of a care coordinator.

**Coercive measures**: refers to all actions that involve forcing patients and includes involuntary medication, seclusion, restraints etc.

**CRPD:** The Convention on the Rights of Persons with Disabilities. A United Nation human rights treaty to protect the rights and dignity of people with disabilities, including disability resulting from mental health conditions. Devised in 2006 and in force since 2008, it has been ratified by 185 countries, but not yet fully implemented in the field of psychiatry.

**Compulsion/compulsory care**: Care that is sanctioned by law in order to address a mental health condition. Can occur in inpatient and outpatient settings.

**Decision-making capacity:** Decision making capacity means the ability to make (treatment) decisions. It is usually assessed by checking that the person is able to understand and retain information, weigh it up to make a choice, and express this. In some jurisdictions, the lack of decision-making capacity is a legal requirement for compulsory care.

**ECT**: Electroconvulsive Therapy. Treatment involving electric currents passing through the brain.

**Independent review body**: A body, independent of the treating clinicians and hospital, created specifically to check that legal criteria for compulsory care are met in individual cases, and deal with complaints and appeals. It usually consists of independent psychiatrists and lawyers.

**Involuntary admission**: a hospital admission sanctioned by law of an individual due to their mental health condition, regardless of his or her will. The law usually specifies certain conditions that must be met for an involuntary admission to be allowed.

**Involuntary admission for observation**: A type of involuntary admission for the purposes of observing whether a patient needs/meets the criteria for involuntary treatment, usually allowed for a short period if certain conditions are met.

**Involuntary treatment**: treatment sanctioned by law to treat an individual’s mental health condition, regardless of his or her will (most often with medication). The law usually specifies certain conditions that must be met for involuntary treatment to be allowed. Can occur in inpatient and outpatient settings

**Involuntary treatment in the community**: refers to various forms of legal regimes available in some countries – such as Community Treatment Orders – that compel individuals to adhere to treatment, certain conditions and/or supervision while they reside in the community. The law usually specifies certain conditions that must be met for involuntary treatment in the community to be allowed.

**Involuntary placement**: The legal status of someone subjected to mental health law. Can apply to both involuntary admission and involuntary treatment in the community.

**Isolation**: refers to the placement of someone admitted in hospital in a specified room where they are separated from other people and from which they are prevented to leave. Includes solitary confinement.

**Least restrictive option**: In most mental health legislation, it is specified that clinicians are obliged to use treatment or a setting for treatment which meets the person’s treatment needs while also imposing the fewest possible restrictions on that person’s rights.

**Mechanical restraint**: refers to the use of a restrictive device, like straps, a set of limb cuffs, belts or jackets to restrict the person’s immediate movement.

**Non-legal advocacy services**: refers to services other than legal services, focused on establishing, protecting and maintaining a person’s rights while in psychiatric services, including rights to choose and refuse treatment, proper communication and due process. Example: Counsellors, social workers, or service user organizations specializing in advocacy.

**Open-area confinement:** refers to the restrictions of someone movement in hospital to a specified area where they are separated from other patients, under close supervision from staff.

**Physical restraints**: refers to physically holding a person (for example, by the arms or on the ground) in order to administer medication or otherwise control the person.

**Proxy consent**: This refers to consent given by a legally appointed representative on behalf of the person unable to give informed consent to a therapeutic intervention.

**Review mechanisms**: refers to a structured and on-going process of review, in individual cases, how legal mechanisms are being practiced, and whether legal criteria are met. Example: Mental health review tribunals

**Seclusion**: refers to the placement of someone admitted in hospital to a separate part of the hospital ward, or in a specified room or secured area from which he or she is prevented from leaving.

**Solitary confinement**, see isolation

**Supported Decision Making**: The process by which a person with a mental health condition is supported to making decisions. This can be structured support by clinical staff or others, to facilitate that the person’s will and preferences are respected in treatment decisions.

**Treatment plan:** A formalized plan for the treatment of a person, tailored to their individual situation and needs.

**FOSTREN LAW AND POLICY PROJECT**

**PART 1: MENTAL HEALTH LEGISLATION**

This part intends to map your country’s legislation related to the treatment of persons with mental disorders. Please note that we are interested in ***the letter of the law*** (how the law is phrased) and not the *spirit* of the law (how it is interpreted). You will, however, have the opportunity to comment where there is a difference between the letter of the law and common interpretation/practice where you consider this to be of importance.

Some relevant official information might found in the Country Profiles of the WHO  Mental Health Atlas 2020 ([www.who.int/teams/mental-health-and-substance-use/data-research/mental-health-atlas](http://www.who.int/teams/mental-health-and-substance-use/data-research/mental-health-atlas)), but this information might need to be checked to see if it is in date.

Please note that all terms marked with and an asterisk (*) are defined in the attached glossary.

**GENERAL ISSUES**

| 1. **Name of country:** | | | | | | | | | |  |  |
| --- | --- | --- | --- | --- | --- | --- | --- | --- | --- | --- | --- |
| 1. **1. Are there specific mental health laws that regulate involuntary placement or treatment of people with mental disorders?** | | | | | | | | | |  |  |
| **☐** | | Yes | | |  |  |  |  |  |  | |
| **☐** | | No | | |  |  |  |  |  |  | |
| **☐** | | *Covered in more general legislation (specify,)* | | | | | | | | | |
|  | | | | | | | | | | |  |
| 1. **2. Please state the year of the last version of the approved mental health act referred above.** | | | | | | | | | | |  |
| Year: | | |  | Name of document: |  | | | | | | |

| 1. **3. Is the legislation available online?** | | | | | | | | |
| --- | --- | --- | --- | --- | --- | --- | --- | --- |
| **☐** | | Yes |  |  |  |  |  |  |
| **☐** | | No |  |  |  |  |  |  |
| *If yes, please provide link.* | | | | | | | | |
|  | | | | | | | | |

| 1. **4. Does the law governing involuntary care exist in English translation?** | | | | | | | | | | |  |
| --- | --- | --- | --- | --- | --- | --- | --- | --- | --- | --- | --- |
| **☐** | | Yes | |  | |  |  |  |  |  |  |
| **☐** | | No | |  | |  |  |  |  |  |  |
| *If yes, please provide the link and specify if this is the latest version of the Act* | | | | | | | | | | |  |
|  | | | | | | | | | | | |
|  | |  | |  | | | | | | |  |

| 1. **5. Does the law specify which group of people may be subject to the law?** | | | | | | | | |
| --- | --- | --- | --- | --- | --- | --- | --- | --- |
| **☐** | | Yes |  |  |  |  |  |  |
| **☐** | | No |  |  |  |  |  |  |
| *If yes, please specify* | | | | | | | | |
| \|  \| \| --- \| | | | | | | | | |

| **6. Does the law define what is meant by mental disorder/mental illness/mental disability?** | | | | | | | | |
| --- | --- | --- | --- | --- | --- | --- | --- | --- |
| **☐** | | Yes |  |  |  |  |  |  |
| **☐** | | No |  |  |  |  |  |  |
| *If yes, please provide the link and specify, Definition:* | | | | | | | | |
|  | | | | | | | | |

| **7. Does the law specify what severity of a mental disorder is required to fulfil the legal criteria?** | | | | | | | |  |
| --- | --- | --- | --- | --- | --- | --- | --- | --- |
| **☐** | Yes |  |  |  |  |  |  | |
| **☐** | No |  |  |  |  |  |  | |
| *If yes, please specify* | | | | | | | | |
|  | | | | | | | | |

| 1. **8. Does the law permit:**    1. **The conversion of a voluntary admission to an involuntary admission?** | | | | | | | | | |  |  |
| --- | --- | --- | --- | --- | --- | --- | --- | --- | --- | --- | --- |
| **☐** | Yes |  | |  |  |  |  |  | |  |  |
| **☐** | No | |  |  |  |  |  |  | |  |  |
| *If yes, please specify* | | | | | | | | | | | |
|  | | | | | | | | | | |  |

| - 1. **The conversion of an involuntary admission to a voluntary admission?** | | | | | | | | |
| --- | --- | --- | --- | --- | --- | --- | --- | --- |
| **☐** | Yes |  |  |  |  |  |  | |
| **☐** | No |  |  |  |  |  |  | |
| *If yes, please specify* | | | | | | | |  |
|  | | | | | | | |  |

| 1. **9. Does the law define “competence” or “capacity”?** | | | | | | | | |  |  |
| --- | --- | --- | --- | --- | --- | --- | --- | --- | --- | --- |
| **☐** | | Yes |  |  |  |  |  |  |  |  |
| **☐** | | No |  |  |  |  |  |  |  |  |
| *If yes, please specify* | | | | | | | | |  |  |
|  | | | | | | | | | | |

| 1. **Does the law specify that involuntary admission/treatment should be only used as a last resort?** | | | | | | | |  |
| --- | --- | --- | --- | --- | --- | --- | --- | --- |
| **☐** | Yes |  |  |  |  |  |  | |
| **☐** | No |  |  |  |  |  |  | |
| 1. **Does the law require that a care/treatment plan* is prepared for people who fulfil the criteria for involuntary admission?** | | | | | | | |  |
| **☐** | Yes |  |  |  |  |  |  | |
| **☐** | No |  |  |  |  |  |  | |

| 1. **If yes, does the law require that one or more practitioner(s) to agree on the treatment plan?** | | | | | | | | | | |
| --- | --- | --- | --- | --- | --- | --- | --- | --- | --- | --- |
| **☐** | | Yes | |  | |  |  |  |  |  |
| **☐** | | No | |  | |  |  |  |  |  |
| *If yes, how many and what level of expertise* | | | | | | | | | | |
|  | | | | | | | | | | |
|  | |  | |  | | | | | | |

| \| 1. **Do patients who are referred to or subjected to involuntary care have the right to free legal representation/free legal aid?** \| \| \| \| \| --- \| --- \| --- \| --- \| \| **☐** \| \| Yes \|  \| \| **☐** \| \| No \|  \|  \| 1. **Do patients have the right to non-legal advocacy services*?** \| \| \| \| \| \| \| \| \| \| \| \| \| \| \| \| \| --- \| --- \| --- \| --- \| --- \| --- \| --- \| --- \| --- \| --- \| --- \| --- \| --- \| --- \| --- \| --- \| \| **☐** \| \| \| Yes \| \|  \| \| \|  \| \| \| \|  \|  \| \|  \|  \| \| **☐** \| \| \| No \|  \| \|  \|  \| \|  \|  \|  \| \| \| \| *If yes, please specify* \| \| \| \| \| \| \| \| \| \| \| \| \| \| \| \| \| \|  \| \| \| \| \| \| \| \| \| \| \| \| \| \| \| \| \| \|  \|  \| 1. **Does the law prohibit patients under involuntary care to be part of research studies?** \| \| \| \| \| \| \| \| \| \| --- \| --- \| --- \| --- \| --- \| --- \| --- \| --- \| --- \| \| **☐** \| \| Yes \|  \|  \|  \|  \|  \|  \| \| **☐** \| \| No \|  \|  \|  \|  \|  \|  \| \| **☐** \| \| Not specified \|  \|  \|  \|  \|  \|  \| \| *If yes, please specify* \| \| \| \| \| \| \| \| \| \|  \| \| \| \| \| \| \| \| \| \| \| |
| --- | --- | --- | --- | --- | --- | --- | --- | --- | --- | --- | --- | --- | --- | --- | --- | --- | --- | --- | --- | --- | --- | --- | --- | --- | --- | --- | --- | --- | --- | --- | --- | --- | --- | --- | --- | --- | --- | --- | --- | --- | --- | --- | --- | --- | --- | --- | --- | --- | --- | --- | --- | --- | --- | --- | --- | --- | --- | --- | --- | --- | --- | --- | --- | --- | --- | --- | --- | --- | --- | --- | --- | --- | --- | --- | --- | --- | --- | --- | --- | --- | --- | --- | --- | --- | --- | --- | --- | --- | --- | --- | --- | --- | --- | --- | --- | --- | --- | --- | --- | --- | --- | --- | --- | --- | --- | --- | --- | --- | --- | --- | --- | --- | --- | --- | --- | --- | --- | --- | --- | --- | --- | --- | --- | --- | --- | --- | --- | --- | --- | --- | --- | --- | --- | --- | --- | --- | --- | --- | --- | --- | --- | --- | --- | --- | --- | --- | --- | --- | --- | --- | --- |

**INVOLUNTARY ADMISSIONS AND TREATMENT**

Involuntary admission (when separate from treatment) and involuntary treatment (where admission and treatment are combined)

| 1. **What are the legal criteria that must be met to allow involuntary admission- (*Tick all that apply)?***  \| **☐** \| Evidence of mental disorder \| \| --- \| --- \| \| **☐** \| Evidence of mental disorder of specified severity, such as psychosis or symptoms with equal impact (if yes, how is it specified) \| \| **☐** \| Serious risk of harm to self or others (if yes, how is it specified?) \| \|  \| \|  \| \| **☐** \| Substantial risk of serious deterioration in the patient’s condition if treatment is not given? \| \| **☐** \| Prospect for substantial improvement in the patient’s condition if treatment is given \| \| **☐** \| The patient lacks decision-making capacity* \| \| **☐** \| Voluntariness has been exhausted/is futile \| \| **☐** \| The patient has been consulted \| \| **☐** \| The admission is in the patient’s best interest \| \| **☐** \| There are no specified criteria \| \| **☐** \| Other criteria – specify \| \|  \| \| |
| --- | --- | --- | --- | --- | --- | --- | --- | --- | --- | --- | --- | --- | --- | --- | --- | --- | --- | --- | --- | --- | --- | --- | --- | --- | --- | --- | --- |

| 1. **Does the law specify that patients must be discharged from involuntary admission as soon as they no longer fulfil the criteria for involuntary admission?** | | | | | | | |  |  |
| --- | --- | --- | --- | --- | --- | --- | --- | --- | --- |
| **☐** | | Yes |  |  |  |  |  |  | |
| **☐** | | No |  |  |  |  |  |  | |
| 1. **Does the law insist on accreditation of a facility before it can admit involuntary patients?** | | | | | | | | | |
| **☐** | | Yes |  |  |  |  |  |  | |
| **☐** | | No |  |  |  |  |  |  | |

| 1. **Is the principle of the least restrictive environment applied to involuntary admissions?** | | | | | | | | |
| --- | --- | --- | --- | --- | --- | --- | --- | --- |
| **☐** | | Yes |  |  |  |  |  |  |
| **☐** | | No |  |  |  |  |  |  |

| 1. **Who is required to certify that the criteria for involuntary admission have been met? ( e.g., two accredited mental health care practitioners, judge?) *Specify*** | |
| --- | --- |
|  | |
|  | |

1. **Who has the authority to admit involuntary? (*tick all that apply*)**

| \| **☐** \| \| The responsible clinician who must be a psychiatrist \| \| \| --- \| --- \| --- \| --- \| \| **☐** \| \| The responsible clinician who can be a general practitioner \| \| \| **☐** \| \| An independent authority (e.g., review body* or tribunal) \| \|  1. **Is there a set timeframe within which the decision to involuntary admit someone must be made after the patient arrives at the hospital?** | | | | | | | | | | | | | | | |  |
| --- | --- | --- | --- | --- | --- | --- | --- | --- | --- | --- | --- | --- | --- | --- | --- | --- | --- | --- | --- | --- | --- | --- | --- | --- | --- | --- | --- | --- |
| **☐** | | Yes | | |  | |  |  | |  |  |  | | | | |
| **☐** | | No | | |  | |  |  | |  |  |  | | | | |
| *If yes, please specify* | | | | | | | | | | | | | | | | |
|  | | | | | | | | | | | | | | | | |
| 1. **Does the law insist that patients must be informed of**      - 1. **The reasons for admission***.* | | | | | | | | | | | | | | | |  |
| **☐** | Yes | |  | | |  | | | |  |  |  |  | |  |  |
| **☐** | No | |  | | |  | | | |  |  |  |  | |  |  |

| - 1. **The rights of appeal?** | | | | | | | | |
| --- | --- | --- | --- | --- | --- | --- | --- | --- |
| **☐** | Yes |  |  |  |  |  |  | |
| **☐** | No |  |  |  |  |  |  | |

| 1. **Does the law insist that the patient’s family must be informed of**      - 1. **The reasons for admission***.* | | | | | | | | |
| --- | --- | --- | --- | --- | --- | --- | --- | --- |
| **☐** | Yes |  |  |  |  |  |  | |
| **☐** | No |  |  |  |  |  |  | |

| - 1. **The rights of appeal?** | | | | | | | | |
| --- | --- | --- | --- | --- | --- | --- | --- | --- |
| **☐** | Yes |  |  |  |  |  |  | |
| **☐** | No |  |  |  |  |  |  | |

| 1. **Does the law insist that the patients legal representative must be informed of**     1. **The reasons for admission***.* | | | | | | | | |
| --- | --- | --- | --- | --- | --- | --- | --- | --- |
| **☐** | Yes |  |  |  |  |  |  | |
| **☐** | No |  |  |  |  |  |  | |

| - 1. **The rights of appeal?** | | | | | | | | |
| --- | --- | --- | --- | --- | --- | --- | --- | --- |
| **☐** | Yes |  |  |  |  |  |  | |
| **☐** | No |  |  |  |  |  |  | |

1. **Does the law provide for a right to appeal an involuntary admission?**

| **☐** | Yes |  |  |  |  |  |  |
| --- | --- | --- | --- | --- | --- | --- | --- |
| **☐** | No |  |  |  |  |  |  |

*If yes, who can this be directed to? (tick all that apply)*

| **☐** | The hospital | |
| --- | --- | --- |
| **☐** | An independent review body* such as a mental health tribunal or mental health judge |  |
| **☐** | Court (for example at a higher level than that which made the order) | |

1. **How frequently can the patient appeal? *Specify***

|  |
| --- |

1. **Does the law include a provision for time-bound periodic reviews of involuntary admission by an independent authority?**

| **☐** | Yes |  |  |  |  |  |  |
| --- | --- | --- | --- | --- | --- | --- | --- |
| **☐** | No |  |  |  |  |  |  |
| *If yes, please specify* | | | | | | | |
|  | | | | | | | |

1. **Does the law specify a duration for involuntary admission orders?**

| **☐** | Yes |  |  |  |  |  |  |  |
| --- | --- | --- | --- | --- | --- | --- | --- | --- |
| **☐** | No |  |  |  |  |  |  |  |
| *If yes, please specify* | | | | | | | |  |
|  | | | | | | | | |

1. **How can it be renewed?** ***Specify***

|  |
| --- |

1. **For how long can it be renewed? *Specify***

|  |
| --- |

1. **Are there separate sections in the law for involuntary admission for the purposes of observation/assessment?**

| **☐** | Yes |  |  |  |  |  |  |  |
| --- | --- | --- | --- | --- | --- | --- | --- | --- |
| **☐** | No |  |  |  |  |  |  |  |
| *If yes, please specify* | | | | | | | |  |
|  | | | | | | | | |

1. **Do the same criteria apply as in ordinary involuntary admission?**

| **☐** | Yes |  |  |  |  |  |  | |
| --- | --- | --- | --- | --- | --- | --- | --- | --- |
| **☐** | No |  |  |  |  |  |  | |
| *If yes, please specify* | | | | | | | |  |
|  | | | | | | | |  |

1. **Is there a stipulated permitted period for an involuntary observation?**

| **☐** | Yes |  |  |  |  |  |  |
| --- | --- | --- | --- | --- | --- | --- | --- |
| **☐** | No |  |  |  |  |  |  |
| *If yes, please specify* | | | | | | |  |
|  | | | | | | |  |

1. **Are there procedures for converting admission for observation to an ordinary involuntary admission?**

| **☐** | Yes |  |  |  |  |  |  |
| --- | --- | --- | --- | --- | --- | --- | --- |
| **☐** | No |  |  |  |  |  |  |
| *If yes, please specify* | | | | | | |  |
|  | | | | | | |  |

**INVOLUNTARY TREATMENT**

1. **Does the law permit the use of involuntary ECT (Electroconvulsive Treatment)?**

| **☐** | Yes |  |  |  |  |  |  | |  |
| --- | --- | --- | --- | --- | --- | --- | --- | --- | --- |
| **☐** | No |  |  |  |  |  |  | |  |
| *If yes, please specify* | | | | | | | |  |  |
|  | | | | | | | | | |

1. **Does the law permit forced nutrition (for instance feeding tubes or intravenous feeding)?**

| **☐** | Yes |  |  |  |  |  |  |  |
| --- | --- | --- | --- | --- | --- | --- | --- | --- |
| **☐** | No |  |  |  |  |  |  |  |
| *If yes, please specify* | | | | | | | |  |
|  | | | | | | | | |

1. **Does the law permit the use of transcranial magnetic stimulation given involuntarily?**

| **☐** | Yes |  |  |  |  |  |  |  |
| --- | --- | --- | --- | --- | --- | --- | --- | --- |
| **☐** | No |  |  |  |  |  |  |  |
| *If yes, please specify* | | | | | | | |  |
|  | | | | | | | | |

1. **Does the law permit surgical procedures such as psychosurgery given involuntarily?**

| **☐** | Yes |  |  |  |  |  |  |  |
| --- | --- | --- | --- | --- | --- | --- | --- | --- |
| **☐** | No |  |  |  |  |  |  |  |
| *If yes, please specify* | | | | | | | |  |
|  | | | | | | | | |

1. **Is the regulation for involuntary treatment separate from that for involuntary admission/placement? (i.e. are separate orders required for admission and for treatment)?**

| **☐** | Yes |  |  |  |  |  |  |  |
| --- | --- | --- | --- | --- | --- | --- | --- | --- |
| **☐** | No |  |  |  |  |  |  |  |
| *If yes, please specify* | | | | | | | |  |
|  | | | | | | | | |

1. **What are the specified criteria that must be met for involuntary treatment?**

| **☐** | Same as for involuntary admission. | | |  |  | | |  |  | |  | |  | | |
| --- | --- | --- | --- | --- | --- | --- | --- | --- | --- | --- | --- | --- | --- | --- | --- |
| **☐** | Yes | | |  |  | | |  | |  | |  |  | | |
| **☐** | No | | |  |  | | |  | |  | |  |  | | |
|  | *If no, tick all that apply:* | | |  |  | | |  | |  | |  |  | | |
| **☐** | Evidence of mental disorder of specified severity | | |  |  | | |  | |  | |  |  | | |
| **☐** | Serious likelihood of harm to self or others | | |  |  | | |  | |  | |  |  | | |
| **☐** | Substantial likelihood of serious deterioration in the patient’s condition if treatment is not given? | | | | | | | | | | | | | | |
| **☐** | Patient lacks capacity to make treatment decisions | | |  |  | | |  | |  | |  |  | | |
| **☐** | Voluntariness has been exhausted/is futile | | |  |  | | |  | |  | |  |  | | |
| **☐** | Therapeutic purposes | | |  |  | | |  | |  | |  |  | | |
| **☐** | There are no specified criteria | | |  |  | | |  | |  | |  |  | | |
| **☐** | other – *specify* | | |  |  | | |  | |  | |  |  | | |
|  | | | | | | | | | | | | | | |  |

1. **Does the law provide for a right to appeal against involuntary treatment?**

| **☐** | Yes |  |  |  |  |  |  |
| --- | --- | --- | --- | --- | --- | --- | --- |
| **☐** | No |  |  |  |  |  |  |

1. **Does the law specify how long involuntary treatment should last?**

| **☐** | Yes |  |  |  |  |  |  |
| --- | --- | --- | --- | --- | --- | --- | --- |
| **☐** | No |  |  |  |  |  |  |
|  | *If yes, please specify* |  | | | | | |

| 1. How can it be renewed? *Specify* |  |
| --- | --- |
|  |  |
|  |  |
| 1. For how long can it be renewed? *Specify* |  |
|  |  |
|  |  |

**FORENSIC TREATMENT IN THE HOSPITAL SETTING**

1. **Does the law specify how long hospital forensic treatment can last?**

| **☐** | Yes |  |  |  |  |  |  |  |
| --- | --- | --- | --- | --- | --- | --- | --- | --- |
| **☐** | No |  |  |  |  |  |  |  |
| *If yes, please specify* | | | | | | | |  |
|  | | | | | | | | |

1. **Does the law provide to the patients the right to refuse medication treatment in forensic care hospital units?**

| **☐** | Yes |  |  |  |  |  |  |  |
| --- | --- | --- | --- | --- | --- | --- | --- | --- |
| **☐** | No |  |  |  |  |  |  |  |
| *If yes, please specify* | | | | | | | |  |
|  | | | | | | | | |

**INVOLUNTARY TREATMENT IN COMMUNITY SETTINGS**

1. **Does the law provide for involuntary treatment in the community* (Community Treatment Order, Mandated Outpatient Treatment, Outpatient Commitment etc)?**

| **☐** | Yes |  |  |  |  |  |  |
| --- | --- | --- | --- | --- | --- | --- | --- |
| **☐** | No |  |  |  |  |  |  |

| 1. **When was this introduced into the legislation? *Specify year*** |  | | | |
| --- | --- | --- | --- | --- |
|  | | | | |
| 1. **What are the criteria for placement on involuntary care in the community?** | | |  | |
| - 1. Same as for involuntary admission? | | |  | |

| **☐** | Yes |  |  |  |  |  |  |
| --- | --- | --- | --- | --- | --- | --- | --- |
| **☐** | No |  |  |  |  |  |  |

- 1. Same but with additional criteria

| **☐** | Yes |  |  |  |  |  |  |
| --- | --- | --- | --- | --- | --- | --- | --- |
| **☐** | No |  |  |  |  |  |  |
| *If yes, please specify* | | | | | | |  |
|  | | | | | | |  |

1. **Can involuntary treatment in the community be ordered when the patient lives in the community or must it follow hospitalization?**

| **☐** | Yes |  |  |  |  |  |  |
| --- | --- | --- | --- | --- | --- | --- | --- |
| **☐** | No |  |  |  |  |  |  |

1. **What does the law oblige all those on involuntary treatment in the community to do?**

***Tick all that apply***

| ☐ | Make themselves available for assessment | | | | | | | |
| --- | --- | --- | --- | --- | --- | --- | --- | --- |
| ☐ | Follow the treatment plan |  |  |  |  |  |  | |
| ☐ | Meet with their clinician regularly |  |  |  |  |  |  | |
| ☐ | Take medication |  |  |  |  |  |  | |
| ☐ | Live in a specified address |  |  |  |  |  |  | |
| ☐ | Other specify |  |  |  |  |  |  | |
|  | | | | | | | |  |

1. **Can conditions be attached to the order that are tailored to the individual?**

| **☐** | Yes |  |  |  |  |  |  | |  |
| --- | --- | --- | --- | --- | --- | --- | --- | --- | --- |
| **☐** | No |  |  |  |  |  |  | |  |
| *If yes, please specify* | | | | | | | | |  |
|  | | | | | | | | | |

1. **Does the law specify how long the order for involuntary treatment in the community can last?**

| **☐** | Yes |  |  |  |  |  |  | |  |
| --- | --- | --- | --- | --- | --- | --- | --- | --- | --- |
| **☐** | No |  |  |  |  |  |  | |  |
|  | *If yes,* *Specify number of days* | | | | | | | | |
|  | | | | | | | | |  |

1. **Can the order for involuntary treatment in the community be renewed?**

| **☐** | Yes |  |  |  |  |  |  |
| --- | --- | --- | --- | --- | --- | --- | --- |
| **☐** | No |  |  |  |  |  |  |

*If yes*,

| 1. **a. For how long can it be renewed? *specify number of days*** | | | | | | | | |  |  |
| --- | --- | --- | --- | --- | --- | --- | --- | --- | --- | --- |
|  | | | | | | | | |  |  |
|  | 1. **b. Are there specific procedures for renewal?** | | | | |  | | | | |
| **☐** | Yes |  |  |  |  |  |  | |  |  |
| **☐** | No |  |  |  |  |  |  | |  |  |
| *If yes, please specify* | | | | | | | |  |  |  |
|  | | | | | | | | | | |

1. **Do the same legal safeguards for involuntary admission apply to involuntary treatment in the community?**

| **☐** | Yes |  |  |  |  |  |  |  |
| --- | --- | --- | --- | --- | --- | --- | --- | --- |
| **☐** | No |  |  |  |  |  |  |  |
| *If yes, please specify* | | | | | | | |  |
|  | | | | | | | | |

**TREATMENT IN THE COMMUNITY OF FORENSIC PATIENTS**

1. **Does the law provide for forensic treatment in the community?**

| **☐** | Yes |  |  |  |  |  |  |  |
| --- | --- | --- | --- | --- | --- | --- | --- | --- |
| **☐** | No |  |  |  |  |  |  |  |
| *If yes, please specify* | | | | | | | |  |
|  | | | | | | | | |

1. **Does the law specify how long forensic treatment in the community can last?**

| **☐** | Yes |  |  |  |  |  |  |  |
| --- | --- | --- | --- | --- | --- | --- | --- | --- |
| **☐** | No |  |  |  |  |  |  |  |
| *If yes, please specify* | | | | | | | |  |
|  | | | | | | | | |

**EMERGENCY SITUATIONS***

1. **Does the law distinguish between emergency situations* and acute situations?**

| **☐** | Yes |  |  |  |  |  |  |
| --- | --- | --- | --- | --- | --- | --- | --- |
| **☐** | No |  |  |  |  |  |  |

1. **Does the law permit involuntary admission and treatment in emergency situations?**

| **☐** | Yes |  |  |  |  |  |  |
| --- | --- | --- | --- | --- | --- | --- | --- |
| **☐** | No |  |  |  |  |  |  |

1. **Are the criteria for emergency admission/treatment limited to situations where there is a high probability of immediate and imminent danger or harm to self and/or others?**

| **☐** | Yes |  |  |  |  |  |  |
| --- | --- | --- | --- | --- | --- | --- | --- |
| **☐** | No |  |  |  |  |  |  |

1. **Does the law specify who is qualified to admit and treat emergency cases?**

| **☐** | Yes |  |  |  |  |  |  |  |
| --- | --- | --- | --- | --- | --- | --- | --- | --- |
| **☐** | No |  |  |  |  |  |  |  |
| *If yes, please specify* | | | | | | | |  |
|  | | | | | | | | |

1. **Does the law specify a time limit for emergency admission (e.g., no longer than 72 hours)?**

| **☐** | Yes |  |  |  |  |  |  |  |
| --- | --- | --- | --- | --- | --- | --- | --- | --- |
| **☐** | No |  |  |  |  |  |  |  |
| *If yes, specify number of hours* | | | | | | | |  |
|  | | | | | | | | |

1. **Does the law specify the need to initiate procedures for involuntary admission and treatment, if needed, as soon as possible after the emergency situation has ended?**

| **☐** | Yes |  |  |  |  |  |  |
| --- | --- | --- | --- | --- | --- | --- | --- |
| **☐** | No |  |  |  |  |  |  |

1. **Do patients, family members and personal representatives have the right to appeal against emergency admission/treatment?**

| **☐** | Yes |  |  |  |  |  |  |  |
| --- | --- | --- | --- | --- | --- | --- | --- | --- |
| **☐** | No |  |  |  |  |  |  |  |
| *If yes, please specify* | | | | | | | |  |
|  | | | | | | | | |

1. **Does legislation for emergency situations allow for the use of ECT without consent?**

| **☐** | Yes |  |  |  |  |  |  |
| --- | --- | --- | --- | --- | --- | --- | --- |
| **☐** | No |  |  |  |  |  |  |

**COERCIVE MEASURES**

1. **Which of the following measures are permitted in the law, and how are these measures defined or described in the law?**
   1. **Seclusion***

| **☐** | Yes |  |  |  |  |  |  |
| --- | --- | --- | --- | --- | --- | --- | --- |
| **☐** | No |  |  |  |  |  |  |
| *Definition if provided*: | | | | | | | |
|  | | | | | | | |

- 1. **Isolation***

| **☐** | Yes |  |  |  |  |  |  |  |
| --- | --- | --- | --- | --- | --- | --- | --- | --- |
| **☐** | No |  |  |  |  |  |  |  |
| *Definition if provided*: | | | | | | | |  |
|  | | | | | | | | |

- 1. **Physical restraints* (holding)**

| **☐** | Yes |  |  |  |  |  |  |  |
| --- | --- | --- | --- | --- | --- | --- | --- | --- |
| **☐** | No |  |  |  |  |  |  |  |
| *Definition if provided*: | | | | | | | |  |
|  | | | | | | | | |

- 1. **Mechanical restraints* (e.g., belts, net beds)**

| **☐** | Yes |  |  |  |  |  |  |  |
| --- | --- | --- | --- | --- | --- | --- | --- | --- |
| **☐** | No |  |  |  |  |  |  |  |
| *Definition if provided, including whether it is specified how the person’s body should be positioned:* | | | | | | | |  |
|  | | | | | | | | |

- 1. **Chemical restraints (short term medication):**

| **☐** | Yes |  |  |  |  |  |  |
| --- | --- | --- | --- | --- | --- | --- | --- |
| **☐** | No |  |  |  |  |  |  |
| *Definition if provided*: | | | | | | | |
|  | | | | | | | |

- 1. **Other,**

| **☐** | Yes |  |  |  |  |  |  |  |
| --- | --- | --- | --- | --- | --- | --- | --- | --- |
| **☐** | No |  |  |  |  |  |  |  |
| ***Please specify*** | | | | | | | |  |
|  | | | | | | | | |

1. **Are there criteria provided by the law for using these coercive measures*?(tick all that apply)**

| **☐** | Seclusion | |  |  |  |  |  |  |  |
| --- | --- | --- | --- | --- | --- | --- | --- | --- | --- |
| **☐** | Isolation |  | |  |  |  |  |  |  |
| **☐** | Physical restraints (holding) | |  |  |  |  |  |  |  |
| **☐** | Mechanical restraints (e.g., belts, net beds) | |  |  |  |  |  |  |  |
| **☐** | Chemical restraints (short term medication): | |  |  |  |  |  |  |  |
| **☐** | *Other* | |  | | | | | |  |
| *Please Specify:* | | | | | | | | |  |
|  | | | | | | | | | |

1. **Does the law specify how long an order for these coercive measures last?** **(tick all that apply)**

| **☐** | Seclusion | |  |  |  |  |  |  |
| --- | --- | --- | --- | --- | --- | --- | --- | --- |
| **☐** | Isolation |  | |  |  |  |  |  |
| **☐** | Physical restraints (holding) | |  |  |  |  |  |  |
| **☐** | Mechanical restraints (e.g., belts, net beds) | |  |  |  |  |  |  |
| **☐** | Chemical restraints (short term medication): | |  |  |  |  |  |  |
| **☐** | *Other* | |  | | | | | |
| *Please Specify:* | | | | | | | | |
|  | | | | | | | | |

1. **Does the law specify if the order for coercive measures* can be renewed?** **(tick all that apply)**

| **☐** | Seclusion | |  |  |  |  |  |  |  |
| --- | --- | --- | --- | --- | --- | --- | --- | --- | --- |
| **☐** | Isolation |  | |  |  |  |  |  |  |
| **☐** | Physical restraints (holding) | |  |  |  |  |  |  |  |
| **☐** | Mechanical restraints (e.g., belts, net beds) | |  |  |  |  |  |  |  |
| **☐** | Chemical restraints (short term medication): | |  |  |  |  |  |  |  |
| **☐** | *Other* | |  | | | | | |  |
| *Please Specify:* | | | | | | | | |  |
|  | | | | | | | | | |

1. **Does the law specify the duty on personnel to supervise the patient during these periods?**

|  | *If Yes,* *which of the coercive measures does this apply to* *(tick all that apply)* | | | | | | | | | |
| --- | --- | --- | --- | --- | --- | --- | --- | --- | --- | --- |
| **☐** | Seclusion | |  |  |  |  |  |  |  |  |
| **☐** | Isolation |  | |  |  |  |  |  |  |  |
| **☐** | Physical restraints (holding) | |  |  |  |  |  |  |  |  |
| **☐** | Mechanical restraints (e.g., belts, net beds) | |  |  |  |  |  |  |  |  |
| **☐** | Chemical restraints (short term medication): | |  |  |  |  |  |  |  |  |
| **☐** | *Other* | |  | | | | | |  |  |
| *Please Specify:* | | | | | | | | |  |  |
|  | | | | | | | | |  |  |

1. **For the use of seclusion and restraints, does the law specify:**
   1. who may authorize it

| **☐** | Yes |  |  |  |  |  |  |
| --- | --- | --- | --- | --- | --- | --- | --- |
| **☐** | No |  |  |  |  |  |  |
| ***If yes, specify***: | | | | | | | |
|  | | | | | | | |

- 1. **What parts of the person’s body can be restrained with mechanical restraints?**

| **☐** | Yes |  |  |  |  |  |  |
| --- | --- | --- | --- | --- | --- | --- | --- |
| **☐** | No |  |  |  |  |  |  |
| ***If yes, specify***: | | | | | | | |
|  | | | | | | | |

- 1. **That accreditation of the facility is needed**

| **☐** | Yes |  |  |  |  |  |  |
| --- | --- | --- | --- | --- | --- | --- | --- |
| **☐** | No |  |  |  |  |  |  |

- 1. **That the reasons and duration of each incident be**
     1. Recorded

| **☐** | Yes |  |  |  |  | |  |  |
| --- | --- | --- | --- | --- | --- | --- | --- | --- |
| **☐** | No |  |  |  |  | |  |  |
| ***If yes, specify***: | | | | | |  |  |  |
|  | | | | | |  |  |  |

- - 1. **Made available to a review board**

| **☐** | Yes |  |  |  |  | |  |  |
| --- | --- | --- | --- | --- | --- | --- | --- | --- |
| **☐** | No |  |  |  |  | |  |  |
| ***If yes, specify***: | | | | | |  |  |  |
|  | | | | | |  |  |  |

.

- 1. **That family members/carers and personal representatives be immediately informed when the patient is subject to seclusion and/or restraint?**

| **☐** | Yes |  |  |  |  |  |  |
| --- | --- | --- | --- | --- | --- | --- | --- |
| **☐** | No |  |  |  |  |  |  |

1. **Does the law specify any procedure for debriefing or other procedures after an episode of coercive measure has ended?**

| **☐** | Yes |  |  |  |  |  |  |  |  |
| --- | --- | --- | --- | --- | --- | --- | --- | --- | --- |
| **☐** | No |  |  |  |  |  |  |  |  |
| ***If yes, specify***: | | | | | | | | |  |
|  | | | | | | | | | |

1. **Does the law prohibit the use of coercive measures* for voluntarily admitted patients?**

| **☐** | Yes |  |  |  |  |  |  |
| --- | --- | --- | --- | --- | --- | --- | --- |
| **☐** | No |  |  |  |  |  |  |

**PROXY CONSENT FOR TREATMENT**

1. **Does the law provide for a person to consent to treatment on a patient’s behalf if that patient has been found incapable of consenting?**

| **☐** | Yes |  |  |  |  |  |  |  |  |
| --- | --- | --- | --- | --- | --- | --- | --- | --- | --- |
| **☐** | No |  |  |  |  |  |  |  |  |
| ***If yes, specify***: | | | | | | | | |  |
|  | | | | | | | | | |

1. **Does the patient have the right to appeal a treatment decision to which a proxy consent* has been given?**

| **☐** | Yes |  |  |  |  |  |  |
| --- | --- | --- | --- | --- | --- | --- | --- |
| **☐** | No |  |  |  |  |  |  |
| **☐** | Not applicable |  |  |  |  |  |  |

1. **Does the law provide for use of “advance directives*”?**

| **☐** | Yes |  |  |  |  |  |  |  |  |
| --- | --- | --- | --- | --- | --- | --- | --- | --- | --- |
| **☐** | No |  |  |  |  |  |  |  |  |
| ***If yes, specify***: | | | | | | | | |  |
|  | | | | | | | | | |

- 1. **How is the term ‘advanced directive’ defined?**

|  | ***Specify***: |  |
| --- | --- | --- |
|  | | |

- 1. **Is the advance directive* legally binding?**

| **☐** | Yes |  |  |  |  |  |  |  |
| --- | --- | --- | --- | --- | --- | --- | --- | --- |
| **☐** | No |  |  |  |  |  |  |  |
| ***If yes, specify***: | | | | | | | | |
|  | | | | | | | | |

- 1. **Does the law make provisions for Supported Decision Making*?**

| **☐** | Yes |  |  |  |  |  |  |  |
| --- | --- | --- | --- | --- | --- | --- | --- | --- |
| **☐** | No |  |  |  |  |  |  |  |
| ***If yes, specify***: | | | | | | | | |
|  | | | | | | | | |

**NON-PROTESTING PATIENTS**

1. **Does the law make provision for patients who lack decision-making capacity* about admission or treatment, but who do not refuse admission or treatment?**

| **☐** | Yes |  |  |  |  |  |  |  |
| --- | --- | --- | --- | --- | --- | --- | --- | --- |
| **☐** | No |  |  |  |  |  |  |  |
| ***If yes, specify***: | | | | | | | | |
|  | | | | | | | | |

- 1. ***If no*, is there legal guidance as to legal safeguards of this group?**

| **☐** | Yes |  |  |  |  |  |  |
| --- | --- | --- | --- | --- | --- | --- | --- |
| **☐** | No |  |  |  |  |  |  |

**OVERSIGHT AND REVIEW MECHANISMS**

1. **Does the law set up a judicial or quasi-judicial body to review processes related to involuntary admission* or treatment and other restrictions of rights?**

| **☐** | Yes |  |  |  |  |  |  |  |  |  |
| --- | --- | --- | --- | --- | --- | --- | --- | --- | --- | --- |
| **☐** | No |  |  |  |  |  |  |  |  |  |
|  | *If yes,* does this body: (*tick all that apply)*   \| **☐** \| Assess each involuntary admission/ treatment? \|  \| \| --- \| --- \| --- \| \| **☐** \| Entertain appeals against involuntary admission and/or involuntary treatment? \|  \|  \| \| **☐** \| Review the cases of patients admitted on an involuntary basis? \|  \| \| **☐** \| Review the cases of patients on long-term voluntary admission? \|  \| \| **☐** \| Regularly monitor patients receiving treatment against their will? \|  \| \| **☐** \| Authorize or prohibit intrusive and irreversible treatments (such as psychosurgery and electroconvulsive therapy, ECT*)? \|  \| | | | | | | | | | |

1. **Does the composition of this body include (*tick all that apply*)**

| **☐** | Legal practitioner |  |  |  |  |  |  |
| --- | --- | --- | --- | --- | --- | --- | --- |
| **☐** | Health care practitioner |  |  |  |  |  |  |
| **☐** | Lay person reflecting the “community” perspective? |  |  |  |  |  |  |
| **☐** | Service users or people with lived experience |  |  |  |  |  |  |

Others *if yes, specify*

|  |
| --- |

1. **Does the law allow for appeal of this body’s decisions to a higher court?**

| **☐** | Yes |  |  |  |  |  |  |
| --- | --- | --- | --- | --- | --- | --- | --- |
| **☐** | No |  |  |  |  |  |  |

**FOSTREN LAW AND POLICY PROJECT**

**PART 2: MENTAL HEALTH POLICY SURROUNDING COMPULSION***

This part asks general questions about your country’s mental health policies. It also seeks information about specific policies related to the use of coercive measures. Other questions relate to positions of stakeholders in your country regarding the use of coercion in mental health care.

The WHO defines a mental health policy as “*an official statement by a government that defines a vision with a set of values, principles and objectives and an overall plan of action to achieve that vision and improve the mental health of a population. The policy should have a detailed plan with concrete strategies and activities that will be implemented with established timelines and the resources needed. Policies and plans for mental health may be standalone or may be integrated into other general health or disability policies or plans. They are considered valid if they have been approved or published by the ministry of health, other line ministries or the country’s parliament*.” [The Mental Health Atlas 2020, Pg. 26]. We add ‘approved by regional health authorities’ to this definition.

Some relevant official information might found in the Country Profiles of the WHO  Mental Health Atlas 2020 ([www.who.int/teams/mental-health-and-substance-use/data-research/mental-health-atlas](http://www.who.int/teams/mental-health-and-substance-use/data-research/mental-health-atlas)), but this information might need to be checked to see if it is in date.

Please note that all terms marked with and an asterisk (*) are defined in the attached glossary.

Where there are more than one relevant policy in the areas asked for, please duplicate the form/table as needed.

**Name of country**:

1. **Is there a regulatory and oversight body to protect the rights of people with mental disorders within and outside mental health facilities?**

| **☐** | Yes |  |  |  |  |  |  |
| --- | --- | --- | --- | --- | --- | --- | --- |
| **☐** | No |  |  |  |  |  |  |
| If yes, specify what body, whether it has national or local jurisdiction | | | | | |  |  |
|  | | | | | |  |  |

|  | Add link to website if available |  |
| --- | --- | --- |

1. **Does the current policy (or law)**
2. Specify that mental health facilities should regularly be inspected

| **☐** | Yes |  |  |  |  |  |  |
| --- | --- | --- | --- | --- | --- | --- | --- |
| **☐** | No |  |  |  |  |  |  |

1. **Provide guidance on how the law should be interpreted (‘code of practice’)**

| **☐** | Yes |  |  |  |  |  |  |  |
| --- | --- | --- | --- | --- | --- | --- | --- | --- |
| **☐** | No |  |  |  |  |  |  |  |
| *If yes ad­d link to the website if available:* | | | | | | | | |
|  | | | | | | | | |

1. **Provide guidance on minimizing compulsory treatments**

| **☐** | Yes |  |  |  |  |  |  |  |
| --- | --- | --- | --- | --- | --- | --- | --- | --- |
| **☐** | No |  |  |  |  |  |  |  |
| *If yes add link to the website if available:* | | | | | | | | |
|  | | | | | | | | |

1. **Direct that registers of accredited facilities or professionals are maintained.**

| **☐** | Yes |  |  |  |  |  |  |  |
| --- | --- | --- | --- | --- | --- | --- | --- | --- |
| **☐** | No |  |  |  |  |  |  |  |
| *If yes add link to the website if available:* | | | | | | | | |
|  | | | | | | | | |

1. **Direct that statistics on the use of involuntary treatment are maintained.**

| **☐** | Yes |  |  |  |  |  |  |  |
| --- | --- | --- | --- | --- | --- | --- | --- | --- |
| **☐** | No |  |  |  |  |  |  |  |
| *If yes add link to the website if available:* | | | | | | | | |
|  | | | | | | | | |

1. **Publish findings on a regular basis?**

| **☐** | Yes |  |  |  |  |  |  |  |
| --- | --- | --- | --- | --- | --- | --- | --- | --- |
| **☐** | No |  |  |  |  |  |  |  |
| *If yes add link to the website if available:* | | | | | | | | |
|  | | | | | | | | |

1. **Has any national/regional policy, been issued from 2005 onwards that is related to the use of involuntary psychiatric care, including compulsion* or coercive measures*? These could relate to more appropriate or reduced use of involuntary care or coercive measures or to the promotion of voluntariness, post-incident reviews or patient’s rights and human rights in mental health care.**

| **Name of issuing body** |  |
| --- | --- |
| **Year issued** |  |
| **Period covered** |  |
| **Title in original language and English translation** |  |
| **Key concerns aims or targets** |  |
| **Motivation for the initiative as described in the document** |  |
| **Reference/link to the document** |  |
| **Reference/link to any known related evaluation, assessment or research** |  |

***--Duplicate form as needed for additional policies***

**Positioning by professional and user organizations**

1. Has any professional body (organization for psychiatrists, psychologists, nurses, ethics committees, other) issued any initiatives, codes of practice or positions on professional ethics from 2005 onwards related to

- The use of involuntary psychiatric care, including coercive measures*. These could relate to more appropriate or reduced use of involuntary care or coercive measures

- The promotion of voluntariness, patient’s rights and human rights in mental health care.

| **Name of issuing body** |  |
| --- | --- |
| **Year issued** |  |
| **Period covered** |  |
| **Title in original language and English translation** |  |
| **Key concerns aims or targets** |  |
| **Motivation for the initiative as described in the document** |  |
| **Reference/link to the document** |  |
| **Reference/link to any known related evaluation, assessment or research** |  |

***--Duplicate form as needed for additional policies***

1. **Have any national or major service user organizations issued any initiatives or statements from 2005 onwards related to**

- The use of involuntary psychiatric care, including coercive measures*. These could relate to more appropriate or reduced use of involuntary care or coercive measures
- The promotion of voluntariness, patient’s rights and human rights in mental health care.

| **Name of issuing body** |  |
| --- | --- |
| **Year issued** |  |
| **Period covered** |  |
| **Title in original language and English translation** |  |
| **Key concerns, demands, aims or targets** |  |
| **Motivation for the initiative as described in the document** |  |
| **Reference/link to the document** |  |
| **Reference/link to any known related evaluation, assessment or research** |  |

***--Duplicate form as needed for additional policies***

1. **Is there mandatory reporting to any authority of the following coercive measures*?**

| **☐** | Seclusion | | |  | | | |  | |  |  | |  |  | | | | |
| --- | --- | --- | --- | --- | --- | --- | --- | --- | --- | --- | --- | --- | --- | --- | --- | --- | --- | --- |
| **☐** | Isolation | | |  | | | |  | |  |  | |  |  | | | | |
| **☐** | Physical restraints (holdings | | | |  | | |  | |  |  | |  |  | | | | |
| **☐** | Mechanical restraints (e.g., belts, net beds) | | | | |  | |  | |  |  | |  |  | | | | |
| **☐** | Chemical restraints (short term medication): | | | | |  | |  | |  |  | |  |  | | | | |
| **☐** | Yes | | | |  | |  | | |  | | |  |  |  |  |  |  |
| **☐** | No | | | |  | |  | | |  | | |  |  |  |  |  |  |
| **☐** | Other?? | | | | |  | |  | |  |  | |  |  | | | | |

1. **Is information on the use of involuntary care and coercive measures available in the public domain?**

| **☐** | Yes |  |  |  |  |  |  |  |  |
| --- | --- | --- | --- | --- | --- | --- | --- | --- | --- |
| **☐** | No |  |  |  |  |  |  |  |  |
| *If yes specify:* | | | | | | | | |  |
|  | | | | | | | | | |

1. **Are post incident reviews, debriefs or similar, used after episodes of coercive measures* (tick one)**

| **☐** | Yes, it is part of routine care |  |  |  |  |  |  |  |
| --- | --- | --- | --- | --- | --- | --- | --- | --- |
| **☐** | In some areas only |  |  |  |  |  |  |  |
| **☐** | No |  |  |  |  |  |  | |

**FUTURE DIRECTIONS**

1. **Are there any developments, that are not yet manifest in law, policy or professional statements in the field that indicate the direction of future developments in the country?**

| **☐** | Yes |  |  |  |  |  |  |  |  |
| --- | --- | --- | --- | --- | --- | --- | --- | --- | --- |
| **☐** | No |  |  |  |  |  |  |  |  |
| *If yes add link to the website if available:* | | | | | | | | |  |
|  | | | | | | | | | |

1. **Public discussion/debate surrounding the CRPD* specify**

|  |
| --- |

1. **Legal change being considered/developed/in the process of being passed in the legislative authority (e.g. Parliament) specify**

|  |
| --- |

1. **Recommendations/Guidelines related to the use of involuntary care and coercive measures* at the regional or national level specify**

|  |
| --- |

1. **White papers specify**

|  |
| --- |

1. **Other specify**

|  |
| --- |

**FOSTREN LAW AND POLICY PROJECT**

**Part 2: SERVICE CONTEXT AND COMPULSORY CARE**

This part intends to map the scope and capacity of mental health services and facilities in your country. It also seeks information about the mental health human resources available, and the rates of involuntary admission rates.

Please note that all terms marked with an asterisk (*) are defined in the attached glossary.

Some relevant official information might found in the Country Profiles of the WHO  Mental Health Atlas 2020 ([www.who.int/teams/mental-health-and-substance-use/data-research/mental-health-atlas](http://www.who.int/teams/mental-health-and-substance-use/data-research/mental-health-atlas)), but this information might need to be checked to see if it is in date.

In official statistics it varies whether incidence rates per 100,000 population is calculated on the basis of a countries full population, or only for those who can be subjected to mental health legislation (often those over 18 years of age). Please specify the basis for the rates provided so that any comparison are appropriate.

It might be difficult to obtain information on some items in some countries. Once your efforts are exhausted, these should be marked as ‘not obtainable’. We appreciate that much of the information will not be available in English, but where it is, a link to the relevant online source (in addition to those in local languages) will be most useful.

**Country**:

1. **What type of service is most used for people with severe mental health problems in your country? (tick one)**

| **☐** | Hospital in-patient services |  |  |  |  | |  |  |  |
| --- | --- | --- | --- | --- | --- | --- | --- | --- | --- |
| **☐** | Hospital out-patient services |  |  |  |  | |  |  |  |
| **☐** | Community-based mental health services (community mental health centres or similar) | | | | |  |  |  | |

1. **What are the latest available statistics on beds in inpatient facilities available per 100.000 population? *Please note of the rate is based on total population numbers or population above the age of 18******/other ‘at risk’***

| Facilities | Number of inpatient facilities per 100,000 population | | Number of hospital beds per 100,000 population | | Please indicate year and source of information (www address is available) |
| --- | --- | --- | --- | --- | --- |
|  | Public | Private | Public | Private |  |
| Mental health units in General hospitals |  |  |  |  |  |
| Standalone mental health hospitals |  |  |  |  |  |
| Forensic mental health units |  |  |  |  |  |
| Forensic mental health hospitals |  |  |  |  |  |
| Community based inpatient beds |  |  |  |  |  |
| Community based supported accommodation |  |  |  |  |  |
| Other (Specify) |  |  |  |  |  |
| National population: *please note if this refer to full population or >18 year olds* |  | | | |  |

1. **What are the latest available statistics on the number of human resources working in mental health (public and or private) services? (Please indicate year and source of information)**

| Mental health human resources working in mental health services | Number of human resources by  profession per 100,000 population  *please note if this refer to full population or >18 year olds* | | Please indicate year and source of information (www address is available) |
| --- | --- | --- | --- |
|  | Public | Private |  |
| Psychiatrists |  |  |  |
| Other medical doctors, not specialized in psychiatry |  |  |  |
| General qualified nurses |  |  |  |
| Qualified nurses in mental health |  |  |  |
| Psychologists working in mental health services |  |  |  |
| Psychotherapists (licensed) |  |  |  |
| Social workers |  |  |  |
| Occupational therapists |  |  |  |
| Total |  |  |  |

1. **What is the latest available statistic on the annual number of patients involuntary admitted per 100,000 population?** *please note if this refer to full population or >18 year olds*

| **☐** | Number, year, Source |  |
| --- | --- | --- |
| **☐** | Not available |  |

1. **What is the latest available statistic on the annual number of patients on involuntary care in the community* per 100,000 population?** *please note if this refer to full population or >18 year olds*

| **☐** | Number, year, Source |  |
| --- | --- | --- |
| **☐** | Not available |  |

1. **Do patients in community care have access to**
   1. **Treatment by multi-disciplinary teams (*tick one*)**

| **☐** | Yes, it is part of routine care |  |  |  |  |  |  |
| --- | --- | --- | --- | --- | --- | --- | --- |
| **☐** | In some areas only |  |  |  |  |  |  |
| **☐** | No |  |  |  |  |  |  |

- 1. **Assertive Community Treatment (ACT, FACT or similar) (*tick one*)**

| **☐** | Yes, it is part of routine care |  |  |  |  |  |  |
| --- | --- | --- | --- | --- | --- | --- | --- |
| **☐** | In some areas only |  |  |  |  |  |  |
| **☐** | No |  |  |  |  |  |  |

- 1. **Care Planning Approaches* (*tick one*)**

| **☐** | Yes, it is part of routine care |  |  |  |  |  |  |
| --- | --- | --- | --- | --- | --- | --- | --- |
| **☐** | In some areas only |  |  |  |  |  |  |
| **☐** | No |  |  |  |  |  |  |

- 1. **Crisis planning (*tick one*)**

| **☐** | Yes, it is part of routine care |  |  |  |  |  |  |
| --- | --- | --- | --- | --- | --- | --- | --- |
| **☐** | In some areas only |  |  |  |  |  |  |
| **☐** | No |  |  |  |  |  |  |

1. **What percentage of total health expenditure in 2019, 2020, 2021 is directed to mental health services? * (Please indicate the year and source of information)**

| Proportion^1^ | Please specify if the  proportion refers to budget or expenditure | Information not  available | Please indicate year and source of information (www address is available) |
| --- | --- | --- | --- |
|  |  |  |  |

^1^Calculated as total amount of money spent for mental health divided by the total amount of money spent by the government (or the mandatory social health insurance) for health (proportion of mental health budget form global health budget)

1. **Not all types of coercive interventions are codified in law. Please indicate which of the following are used in mental health services in your country whether or not they are mentioned in the law. *Tick all that apply***

| **☐** | Seclusion |  |  |  |  |  |  |
| --- | --- | --- | --- | --- | --- | --- | --- |
| **☐** | Open area confinement* |  |  |  |  |  |  |
| **☐** | Isolation |  |  |  |  |  |  |
| **☐** | Net beds |  |  |  |  |  |  |
| **☐** | Cage beds |  |  |  |  |  |  |
| **☐** | Intensive care area |  |  |  |  |  |  |
| **☐** | Constant observation |  |  |  |  |  |  |
| **☐** | Formal close observation (not constant) |  |  |  |  |  |  |
| **☐** | Mandated time out |  |  |  |  |  |  |
